# Associations Between Practices and Behaviors at the Health Facility Level and Supply Chain Management for Antiretrovirals: Evidence from Cameroon, Namibia, and Swaziland

**DOI:** 10.9745/GHSP-D-19-00063

**Published:** 2019-06-24

**Authors:** Diana Bowser, Laura Krech, David Mabirizi, Angela Y. Chang, David Kapaon, Thomas Bossert

**Affiliations:** a Institute for Global Health, Heller School for Social Policy and Management, Brandeis University, Waltham, MA, USA.; b Global Public Health Consultant, Ada, MI, USA.; c Management Sciences for Health (MSH), Arlington, VA, USA.; d Institute for Health Metrics and Evaluation, University of Washington, Seattle, WA, USA.; e Heller School for Social Policy and Management, Brandeis University, Waltham, MA, USA.; f T.H. Chan School of Public Health, Harvard University, Boston, MA, USA.

## Abstract

**Background:**

While measuring, monitoring, and improving supply chain management (SCM) for antiretrovirals (ARVs) is understood at many levels of health systems, a gap remains in the identification and measurement of facility-level practices and behaviors that affect SCM. This study identifies practices and behaviors that are associated with SCM of ARVs at the hospital level and proposes new indicators for measurement.

**Methods:**

We performed an in-depth literature review to identify facility-level practices and behaviors and existing indicators that are associated with SCM. We used the United States Agency for International Development's 2013 National Supply Chain Assessment Toolkit to define 7 supply chain function areas to frame the study. Qualitative, semistructured key informant and focus group interviews were conducted in hospitals with health professionals from Cameroon, Namibia, and Swaziland to understand facility-level practices and behaviors.

**Results:**

Using the results from 54 key informant and focus group interviews from 12 hospitals, we identified 30 practices and behaviors that may affect ARV SCM at the facility level. The following practice areas were particularly associated with SCM: order verification, actions taken when ARV stock is received, changes in prescription and dispensing due to ARV stock-out, actions to ensure patient adherence, and communication with other affiliated facilities and higher-level SCM. We subsequently developed measurable indicators for future research.

**Conclusion:**

This study characterizes facility-level practices and behaviors that can affect ARV SCM. It also identifies gaps in their measurement. While this study uses ARVs as a tracer medicine to understand gaps in practices at the facility level, many of the findings are more broadly applicable to other medicines in an integrated setting. This study provides real-world evidence and the groundwork for further research to characterize the link between 30 facility-level practices and behaviors and ARV SCM at the facility and central levels.

## Abstract (precis)

Using antiretrovirals (ARVs) as tracer products, we identified the following key practices that may affect supply chain management at the facility level: order verification, actions taken when stock is received, changes in prescription and dispensing due to ARV stock-out, actions to ensure patient adherence, and communication with other affiliated facilities and higher-level supply chain management. We propose a set of indicators to measure these practices.

## BACKGROUND

Scaling up of antiretroviral (ARV) therapy in low- and middle-income countries, especially in Africa, has been supported by 2 main sources: the U.S. President's Emergency Plan for AIDS Relief (PEPFAR) and the Global Fund to Fight HIV/AIDS, Tuberculosis and Malaria (Global Fund).^1^ Many developing middle-income countries use their domestic resources to varying degrees to contribute to antiretroviral therapy (ART) scale-up. During the past decade, countries receiving donor support for HIV/AIDS programs have been transitioning disease-specific supply chains into integrated, essential medicine supply chains with domestic financing and government support.^2^ Some countries have accomplished this by increasing Ministry of Health (MOH) staff and funding budgets on par with donor organizations.^3^ Despite these efforts, supply chain management (SCM) remains a weak part of the national health system in many countries, where the delivery of ARVs is not yet sustainable or fully transitioned to the MOH, leaving the risk of stock-out for ARVs high.^2^

Effective and efficient communication between central, regional, and local health facility levels as well as quantification, secure transport, and quality assurance of medical products are all required parts of a functional supply chain.^4^^–^^7^ Although much is understood about how to measure, monitor, and improve ARV SCM at the central and regional levels of a health system, less has been done to identify and measure facility-level practices and behaviors that can affect SCM.^5^^,^^6^^,^^8^^–^^12^ In this article, we define facility-level SCM as activities related to managing and ordering inventory and monitoring the performance of facilities at the point of service delivery carried out by health care providers, managers, and administrators involved in prescribing and dispensing.^7^ These facilities may include public and private hospitals and health facilities, clinics, pharmacies, drug stores, and other outlets. Practices are defined as actions taken (or lack of actions taken) within facilities by health care workers and/or managers that affect SCM. Behaviors are defined as the leadership or management styles as well as the quality of relationships and communication between health care workers and managers at the facility level and those working in higher-level SCM management positions.

Comprehensive, well-designed reports and tools already exist with measurable indicators across various aspects of the supply chain. They include Management Sciences for Health's (MSH) *Managing Drug Supply-3* (MDS-3): *Managing Access to Medicines and Health Technologies*; *Logistics Indicator Assessment Tool* (LIAT), which was developed with support from the United States Agency for International Development (USAID); the *Logistics Handbook* (USAID); and USAID's *National Supply Chain Assessment Toolkit* (NSCA), which includes the *Capability Maturity Model Diagnostic Tool* and *National Supply Chain Key Performance Indicator Assessment* (USAID).^2^^,^^13^^–^^16^ However, most of these reports and tools do not clearly distinguish or link between central, regional, and facility-level indicators. For example, no clear evidence exists that indicators developed and validated at the central and regional levels can be used for measuring SCM at facility levels. In addition, these tools cannot be readily used to understand how established facility-level SCM indicators affect and are connected to those at the regional and central levels. Furthermore, only a few studies examine associations of facility-level practice indicators to SCM.^17^^–^^19^

Some facility-level indicators of practices and behaviors do exist, but many of them have not been shown to be associated with SCM, and no system is in place to measure and monitor them in a systematic way.^2^^,^^15^^,^^20^ In this study, we aimed to identify facility-level practices and behaviors and their associations to ARV SCM and to propose indicators to measure these practices and behaviors in future studies.

We aimed to identify facility-level practices and behaviors and their associations to ARV supply chain management.

This study is unique in 2 ways. As previously stated, few studies have tried to expand understanding of facility-level practices and behaviors that could affect ARV SCM.^2^^,^^6^^,^^20^^,^^21^ Second, very limited publications or reports utilize a qualitative methodological framework of analysis to elucidate what is currently happening at the facility level as a means of determining the practices and behaviors that may have an impact on facility-level SCM.^22^

## METHODS

We used a mixed research methodology that incorporated both quantitative and qualitative methods. The methodology included 4 phases consisting of a literature review, country and health facility selection and survey instrument development, in-country data collection, and data synthesis and analysis.

### Literature Review

First, we conducted an in-depth review of published and gray literature to identify facility-level SCM indicators that can be used to measure and categorize facility-level practices and behaviors. The literature review was performed to identify facility-level practices and behaviors with evidence for an impact on SCM, with a specific focus on availability of and access to HIV/AIDS commodities. We focused mainly on literature from low- and middle-income countries, but we also searched for management literature in business journals, which included sources from higher-income countries. Key terms used in the search were HIV/AIDS, medicines and/or pharmaceutical supply chain management, supply chain performance, supply chain performance measurement, qualitative HIV/AIDS studies, behaviors and/or practices that impact supply chain, HIV/AIDS supply chain guidelines, and terms related to each step of the supply chain (e.g., product selection, forecasting, procurement, warehousing, inventory management, transportation, prescribing, and dispensing).

The review began with a search of published materials from 1990 to 2015 in the following databases: PubMed, Web of Science, and Google Scholar. We reviewed official reports published by nonprofit organizations, development agencies, and their implementing partners, in particular, USAID, MSH, John Snow Inc., and the World Health Organization.^5^^,^^6^^,^^8^^,^^9^^,^^23^^–^^25^ The following tools and reports were also analyzed: *Managing Access to Medicines and Health Technologies* (MDS-3, MSH), the *Logistics Indicators Assessment Tool* (USAID), *Logistics Handbook* (USAID), the 2013 toolkit documents from USAID's NSCA: *Capability Maturity Model Diagnostic Tool* and *National Supply Chain Key Performance Indicator Assessment.*

To identify existing facility-level SCM indicators, we reviewed indicators related to product selection, forecasting and supply planning, procurement, warehousing and inventory management, transportation, dispensing, waste management, laboratory issuing, information management, infrastructure, human resources, demand factors, behaviors and practices, and perceptions related to HIV/AIDS treatment.^5^^,^^8^^,^^17^^,^^24^^,^^26^^,^^27^ In addition to facility-level indicators, supply chain function areas were also reviewed in the literature. However, after analyzing numerous supply chain tools and reports, we chose to extract and adapt the supply chain function areas from USAID's 2013 NSCA Toolkit to categorize the facility-level practices and behaviors identified in the literature review. The main rationale for this decision was that the majority of the supply chain indicators and function areas proposed in that toolkit originated from and consolidated various aspects of other major tools.

The final 7 supply chain function areas used to classify facility-level behaviors and practices related to ARV SCM were forecasting and quantification; warehousing and inventory management; prescribing and dispensing; communication; information management; infrastructure; and human resources.

7 supply chain function areas were used to classify facility-level behaviors and practices related to ARV SCM.

The Figure shows the theoretical framework that guided the in-depth literature review, the methods, and the analysis. The blue boxes highlight areas included in the scope of the study. The framework shows how the study focused on identifying facility-level practices and behaviors, within facility-level supply chain function areas, and then linked them with measurable indicators. This framework also demonstrates how facility-level practices and behaviors connect to facility- and central-level SCM outcomes. We also considered health system design factors such as human resources, organization, and leadership. The orange boxes are included to capture the entire system, but they are not part of this study.

**FIGURE:**
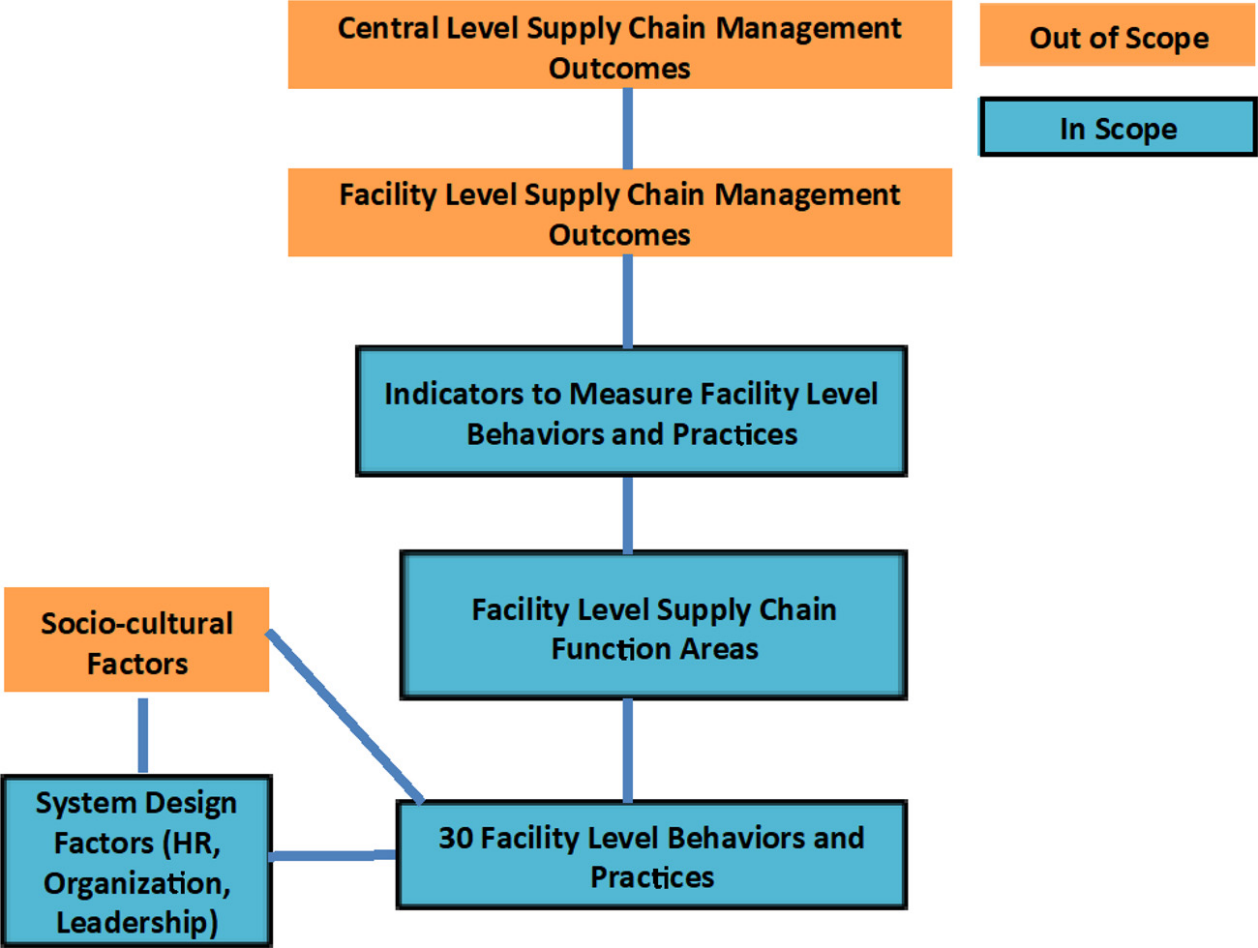
Study Design Focuses on the Practices and Behaviors at the Health Facility Level Abbreviation: HR, human resources.

### Country and Health Facility Selection and Survey Instrument Development

Country selection was carried out in coordination with the USAID-funded Systems for Improved Access to Pharmaceuticals and Services (SIAPS) Program, implemented by MSH. Countries selected were PEPFAR-funded African countries based on the source of funding for this research study.

The additional selection criteria pertained to language spoken (at least 1 francophone and 1 anglophone country), geographical variation (west/south/east), and availability and preparedness of country teams. Based on these criteria, 1 francophone and 2 anglophone countries were selected that also represented varied geographic areas: Anglophone/West/Southern Africa (Namibia), Anglophone/South/Eastern Africa (Swaziland), and Francophone/West/Central Africa (Cameroon). Further information was gathered based on each country selected, including a desktop review of the official, published, and gray literature on the ARV supply chain in each country.

We collaborated with local country experts from the MOH, particularly managers of national HIV/AIDS programs and SIAPS colleagues, to select the health facilities included in the study. Country experts assisted the study team in obtaining further reports and data that were not available publicly or online. Due to the variation and different types of lower-level facilities (health posts, clinics, etc.) in all 3 countries, hospitals were chosen as the facility unit of analysis due to more standard patient care responsibilities, availability of pharmacies, and types of health care workers and human resources. With technical assistance from national HIV/AIDS program managers, 4 hospitals in different geographic areas from each country were identified, for a total sample size of 12 hospitals.

4 hospitals in different geographic areas from each country were identified, for a total sample size of 12 hospitals.

The hospitals included in the study were selected based on indicators that we reviewed with each collaborating country study team. The indicators used to select the hospitals (listed below) were consistent within a country, but varied across the 3 countries. This variation was due to the 3 countries having different health information systems, which led to facilities across countries having different comparable and available indictors. The following 5 supply chain–related indicators were chosen to select facilities in **Namibia**:^28^^,^^29^

- Storage condition: cleanliness, tidiness, appropriate arrangement of pharmaceuticals, and temperature monitoring practices
- Inventory management and quantification: stock card use, cold chain management, and interim orders
- Use of the Electronic Dispensing Tool for stock management (such tools help maintain basic patient profile information, medicine history, and other data that are essential for the dispenser to know at the time of dispensing)
- Completeness of ART reports submitted to the Ministry of Health and Social Services (MOHSS)
- Availability of adequate ART stock on hand

The following 2 supply chain–related indicators were used in **Cameroon**:

- Discrepancies between patient figures and stock records
- Discrepancies between physical stock count and stock records

The following supply chain–related indicator was used in **Swaziland**:

- Months of stock on hand

Based on the in-depth literature review as well as country protocols for the management of HIV/AIDS patients in the participating countries, we developed qualitative interview guides to identify the practices and behaviors at the facility level that could have an impact on ARV SCM. Facility-level practices and behaviors were included in the guide for the following activities: prescribing, counseling, dispensing, clinical services, data-generating activities, data analysis, data reporting, forecasting, ordering, handling of expired stock, behaviors if a stock-out happens, submission of reports and orders, placing emergency orders, communication within and between facilities, and more general practices and behaviors regarding management leadership style and the quality of communication between facilities and higher-level SCM. All these activities fell within the 7 supply chain function areas.

Interview guides were developed to facilitate focus group discussions with key personnel at the facility level and interviews with key informants at central health system levels (e.g., the MOH, HIV/AIDS National Program, Central Medical Stores [CMSs]). We identified relevant interviewees based on their reported assigned tasks and daily responsibilities in each of the health facilities in the provision of ARVs and HIV services. We did not rely solely on using job titles or job descriptions because they do not necessarily correspond to actual day-to-day responsibilities and were not consistent across facilities.

### In-Country Data Collection

One-week country visits occurred in January and February of 2014. Two of the authors, both from academia, conducted the interviews solely for this study in order to reduce any bias from donor-related activities, assessments, or evaluations.

First, key informant interviews were conducted with broader health system representatives from the MOH, HIV/AIDS National Program and regional offices, CMSs, and the World Health Organization. Second, key personnel focus group interviews were conducted with health care workers, pharmacists, and managers who interact with patients with HIV/AIDS arriving to the facility (hospital) for HIV medications. No key personnel were key informants.

### Data Synthesis and Analysis

Interviews were recorded through interviewers' notes and later transcribed. A rubric of all practices and behaviors was developed to consistently identify themes and outliers in each of the key practices and behaviors within and across each country. A qualitative thematic analysis was performed to identify patterns in the practices and behaviors at the facility level that could have an impact on ARV SCM. A hybrid of deductive and inductive coding was used; meaning information identified from the literature at the beginning of the study was used as a starting point for analysis (deductive), and new themes and codes later emerged from the country-level data analysis of the interviews (inductive). All facility-level practices and behaviors observed in the countries were mapped to the 7 supply chain function areas adapted from the USAID's NSCA Toolkit.

Due to the extensive amount of qualitative results on all 30 practices and behaviors analyzed, we selected and summarized qualitative information for 7 of them in the results. We selected these 7 because they provide clear examples of how behavior and practice vary between and within countries across the supply chain function areas. In addition, the in-depth literature review and individual country results revealed that these 7 practices and behaviors had an association with SCM.

We selected and summarized qualitative information for 7 particularly relevant practices and behaviors.

The same 2 authors who carried out the in-country interviews and focus groups also carried out the data synthesis and analysis. Data synthesis and analyses were reviewed independently by authors 1 and 4 using a rubric of all identified practices and behaviors. For the small number of cases of disagreements, authors 1 and 4 discussed and resolved the discrepancy together. If the disagreement could not be resolved, author 2 was brought in and resolution was achieved by majority.

All major SCM measurement tools and relevant literature were then reviewed for a final time to identify indicators that mapped to each of the observed facility-level practices and behaviors. All indicators that were identified for each facility-level behavior and practice were listed. For those with no identifiable indicator, we used the results of the in-country interviews to propose new indicators. It should be noted that while this study used ARVs to understand gaps in practices at the facility level, many of the findings can be applied more broadly to medicines in an integrated setting.

### Country Context

Table 1 summarizes relevant information on country context for Cameroon, Namibia, and Swaziland with respect to spending on HIV/AIDS, HIV prevalence, and demographics. The Namibian government has made the largest investment in HIV/AIDS spending, with 64% of the total HIV/AIDS budget coming from the public sector in 2014. Swaziland covers 38% of HIV/AIDS program costs with government funds, and Cameroon spends 15%. Consequently, Cameroon had the largest foreign donor investment in HIV/AIDS in 2014 (70%–80% of HIV/AIDS funding), with most of it coming from the Global Fund. Of the 3 countries, Cameroon has the lowest HIV prevalence, at 4.8% of 15–49-year-olds. Swaziland has one of the highest global HIV prevalence rates, with 26% of 15–49-year-olds living with HIV.

**TABLE 1.** Country Context

	Namibia^30^^,^^31^	Cameroon^32^^,^^33^	Swaziland^34^^,^^35^
**National HIV/AIDS financing in 2014**			
Government spending on HIV/AIDS, % of total HIV/AIDS spending	64	15	38
Foreign donor spending on HIV/AIDS, % total HIV/AIDS spending^a^	23	70–80	60
Private sector spending on HIV/AIDS, % total HIV/AIDS spending	1	10	1.8
**HIV/AIDS prevalence and demographics in 2014**			
HIV/AIDS prevalence among adults ages 15–49, %	16	4.8	26
No. of people living with HIV ages 15–49, thousands	260	660	210
Population, millions	2.3	24	1.4

a Foreign donors include the U.S. President's Emergency Plan for AIDS Relief (PEPFAR), the Global Fund, NGOs, and United Nations agencies. PEPFAR and the Global Fund provide the largest percentage of foreign donor funds in all countries.

Namibia, the largest country in the study, has a population of only 2.3 million people, making it one of the least populated countries in the world. Namibia not only has a shortage of health care workers but also a shortage of supply chain personnel to work in the public health supply chain.^18^ The MOHSS oversees 14 regions with 350 public health facilities and manages the CMS distribution systems in Windhoek, Rundu, and Oshakati (the latter 2 being multiregional medical depots [MRMDs]).^36^ The distribution of medicines is carried out by CMS Windhoek to public health facilities in nearby central and southern Namibia and to the distant MRMDs of Rundu and Oshakati to cover a wide swath of Namibia's territory. MRMDs and public health facilities deliver medicines to health centers, clinics, and hospitals. Namibia operates an integrated pharmaceutical supply chain; ARVs do not have a parallel system of delivery, thus they are distributed with all other medicines from CMSs to clinic levels.

Cameroon is about half the size of Namibia but its population is tenfold higher (23.4 million inhabitants). The National AIDS Commission manages Cameroon's HIV/AIDS program. The national program manager is responsible for all programmatic aspects related to quantification and forecasting of ARVs and implementation of HIV/AIDS policies, among other duties. Cameroon has 10 regional medical stores (RMSs; centres d'approvisionnement pharmaceutiques des regionaux [CAPRs]) in each of its 10 administrative regions. CAPRs deliver medicines to health facilities and all receive ARV stock procured by Cameroon's CMS based in Yaounde (Centrale Nationale d'Approvisionnement en Médicaments Essentials et Consommables Médicaux).^37^ CAPRs distribute ARVs to the 124 ART sites based on health clinic needs; however, when ARVs are only available in limited quantities or facing stock-outs at the national level, ARVs are rationed to health facilities.

Swaziland is a small country with a population of 1.4 million, making it the most densely populated country in this study. Swaziland has an integrated pharmaceutical supply chain for medicines and medical supplies. CMSs are responsible for the SCM of all health commodities including medicines in the public sector. ARVs are directly distributed from CMSs on a monthly schedule to 45 hospitals and health facilities. ARVs are further delivered down the supply chain from hospitals and health facilities to what are known as baby (feeder) clinics.

### Ethical Approval

The study was approved by the Institutional Review Board (IRB) of the Harvard T.H. Chan School of Public Health (protocol number IRB13-3167) and was determined to be exempt from obtaining written informed consent. The IRB determined that the protocol meets the criteria for exemption per the regulations found at 45 CFR 46.101(b)(2) and any additional review by the IRB was not required. In country, no local IRB approval was required; however, the research team obtained written authorization to conduct the study from the MOH of Cameroon, Namibia, and Swaziland. All participants in this study were adults (≥18 years). The purpose of the study was explained to participants, and verbal informed consent was obtained from all individuals participating in individual and focus group qualitative, semistructured interviews.

## RESULTS

In total, 52 semistructured key informant and personnel interviews were conducted across the 12 hospitals included in the study. The number of key informants and personnel that were interviewed in each hospital varied depending on how many individuals were available on that particular day. For example, in Namibia across the 4 health facilities visited, 8 key informant/personnel interviews were conducted for an average of 2 individuals in each hospital. There were more individuals available for key informant interviews at the broader health system level in Namibia and Cameroon than in Swaziland. Table 2 details the number of key informant interviews conducted in each country and the type of interview.

**TABLE 2.** Semistructured Key Informant and Personnel Focus Group Interviews

Interviews	Namibia	Cameroon	Swaziland
Broader health systems interviews	12	13	1
Hospital interviews (staff involved in HIV/AIDS services and ARV management)	8	12	6
Total interviews (N=52)	20	25	7
Total facilities in the study (N=12)	4	4	4

Abbreviation: ARV, antiretroviral.

52 semistructured key informant and personnel interviews were conducted across 12 hospitals in 3 countries.

Table 3 shows the 30 facility-level practices and behaviors that were identified through key informant and personnel interviews and mapped to 7 facility-level supply chain function areas. Some of these practices and behaviors are activities that are closely linked with routine activities for that particular supply chain function area and the literature supports a link between better completion of this activity and improved SCM. For example, calculation of minimum and maximum (min-max) buffer stock is an activity that is practiced widely across many facilities as part of forecasting and quantification exercises, although specific types of calculation differ. The MOH and donors often provide trainings on the different calculations that can be used to verify and document the maximum and minimum number of months of stock that should remain on the shelf, which should lead to lower stock-outs.^7^

**TABLE 3.** The 30 Supply Chain Management Practices and Behaviors Identified in the Analysis

Facility-Level Supply Chain Function Area and Practice/Behavior	Description of Best Practices From the Literature and Observed Practices From This Study
**Forecasting and Quantification**	
1. Calculation of minimum and maximum buffer stock	Calculation of the minimum and maximum levels of pharmaceutical stock needed over a specified time period, taking into consideration buffer stock, stock used during lead time, and order quantify for one supply period.^5^
2. Use of electronic systems	Use of electronic systems assist in the tracking of services and products delivered to patients. Furthermore, such systems also help to fulfill new monthly orders and maintain stock records, while also assisting in reporting such records to higher-level offices.
3. Use of national guidelines as reference for estimation of needs and reporting	Use of guidelines in inventory control improves poorer performance of logistic systems.^38^
4. Order verification before submission to the central/regional level^a^	A health facility staff member rechecking the ARV requisition request (verifying calculation, order, and inventory stock) before an order is sent to the central or regional level leads to fewer order verification errors.
5. Order fill rate calculation	Order fill rate should be calculated to cut down number of emergency and/or unfilled orders.
6. Late ordering of medicines	Staff should be consistently aware of order dates and treat them as a potential problem so as to avoid late orders
7. Frequency of issuing emergency orders	A study in Mali found that emergency orders of stock are required as facilities receive only about 25% of what they request.^39^ Emergency orders were not reported as frequent or an issue.
**Warehousing and Inventory Management**	
8. Actions taken when stock received from CMS/RMS^a^	Any newly received or issued products are recorded in stock-keeping records. Entries are further updated either when stock is counted during a physical inventory, or as soon as a loss is noticed.^5^
9. Control of access to stock	Security, monitoring, and auditing are some of the methods to prevent stock-outs and losses.^26^
10. Decision on whether to redistribute short-dated stock	Redistribution of short-dated stocks increases the complexity of the supply chain and miscommunication of stock levels between facility and central levels.
11. Location and condition of storage (whether all in one place or separate rooms)	Good inventory control includes appropriate storage space, stock rotation, stock arrangement, cleanliness, security, and fire prevention.^26^
12. ARVs stored separately from other medicines	Due to funding requirements, many ARVs are stored in separate storage areas from other medicines. Access of staff to ARVs is limited as well to prevent theft and diversion.
13. Assigning responsibility of inventory management tasks	In most facilities, a trained nurse, pharmacy assistant, or pharmacist is assigned to manage ARVs. In some facilities a schedule and description of tasks for staff is available and implemented.
14. Frequency of balancing stocks (checking stock cards vs. physical count)	Stock status of each product in storeroom should be assessed regularly (monthly) by staff, comparing the quantities on hand with the quantities that have been entered in inventory control cards.^5^
**Prescribing and Dispensing**	
15. Change in ARV prescription during stock-out^a^	SOPs are needed for the prescribing process in the event of stock-outs to standardize actions among prescribers.
16. Change in dispensing of ARVs during stock-out^a^	Written SOPs are recommended to improve consistency and quality of the dispensing process.^26^ SOPs are needed to standardize actions during the dispensing process.
17. Actions to ensure patient adherence (e.g., pill count)^a^	SOPs are needed for monitoring adherence (e.g., whether to perform pill counting) to ARVs.
**Communication**	
18. Communication within the pharmacy team	A positive team dynamic can be achieved via regularly scheduled weekly/biweekly internal meetings.
19. Communication within the facility	Active communication between pharmaceutical and nonpharmaceutical staff regarding shortages and stock-outs is recommended to increase consistency and accurate recording of prescriptions.
20. Communication with higher-level supply chain management^a^	Improved facility-level SCM performance can be achieved more easily via robust relationships with the regional and central personnel.
21. Communication with affiliated facilities^a^	An increased in accurate reporting and forecasting at the main facility is a potential byproduct of positive and regular communication with any and all affiliated facilities.
22. Communication with hospital executives	Key informants report that direct lines of communication between pharmaceutical staff and hospital executives is recommended to address and avoid shortages and stock-outs.
**Information Management**	
23. Interaction between clinical and dispensing/stock systems	Most facilities do not have linkage between clinical and dispensing information systems. Swaziland does have linked systems and key informants report frequent backlogs on prescription input.
**Infrastructure**	
24. ARV clinic/pharmacy separate from main pharmacy	ARV clinic/pharmacy was observed to be separate from the main pharmacy in some facilities and integrated with others.
**Human Resources**	
25. Training on stock management	An individual's technical ability, personality, and position within the supply chain had a significant impact on supply chain performance.^40^
26. Leadership/management style of the pharmacy	Key informants reported multiple leadership/management styles of the pharmacies. Some were managed/led by regional and senior level pharmacists, others by pharmacists, pharmacist assistant physicians or nurses. Consistent management organization and leadership across pharmacies can improve supply chain performance.
27. Leadership management style of the clinic	Key informants reported that clinics were managed/led by physicians who attend HIV patients and other patients.
28. Attitude to workload of pharmacy staff	Pharmacist assistants and nurses in some facilities reported that workloads were too high, leading to unfinished daily activities, including those linked to supply chain management.
29. Guidelines for providers in the event of a stock-out	There are no standardized guidelines for providers for what to do in the event of a stock-out.
30. Implementation of policies on prescribing and dispensing	Some key informants reported having clear policies of not allowing patients to leave without any medicines.

Abbreviations: ARV, antiretroviral drug; CMS, central medical store; RMS, regional medical store; SCM, supply chain management; SOP, standard operating procedure.

a These practices and behaviors are associated with SCM more than others and are described in detail in the results section.

Other identified practices and behaviors are not routinely carried out as part of that function area, and the literature contains minimal evidence on the link between them and any type of improved SCM. For example, communication between pharmacy team members and the clinical staff dispensing the medications is routinely practiced to different degrees of effectiveness across all the facilities included in the study. However, the literature includes little documentation on these types of communications and the relationship of communication with SCM.

In order to shorten the length of the full qualitative results, we selected 7 of the 30 practices and behaviors associated with ARV SCM as clear examples of the variation in supply chain function areas within each country (Table 4). These practices and behaviors are order verification before submission to the central/regional level; actions taken when stocks received from CMS/RMS; communication with higher-level SCM; communication with affiliated facilities; change in prescription during stock-out; change in dispensing during stock-out; and actions to ensure patient adherence. Additional qualitative information on all 30 practices and behaviors can be found in the SIAPS report *Facility Level Practices and Behaviors That Affect the Performance of the Supply Chain of Antiretroviral Medicines.*^7^ The 7 practices and behaviors reported and discussed in the current study describe what health care workers actually do (what they describe doing) and whether or not it is in line with guidelines for receiving a shipment, making an order, communicating among staff, and managing medicines and commodities.

**TABLE 4.** Selected Facility-Level Practices/Behaviors, Existing Indicators, and Potential New Indicators for Future Testing and Piloting

Facility-Level Supply Chain Function Area	Practice/Behavior	Sample Measurable Indicators From Existing Research and Literature	Potential New Facility-Level Indicators
Forecasting and quantification	Order verification before submission to the central/regional level	Formal work plan and/or schedule for quantification^26^ Average order entry time and order entry accuracy^9^	Orders are verified by staff prior to sending Second-stage order verification by staff member other than the person who filled the order
Warehousing and inventory management	Actions taken when stocks received from CMS/RMS	Average put-away accuracy and put-away time^9^	Immediate shelving of stock upon arrival by appropriate staff member Verification of stock arrival and shelving procedures Reported discrepancies between what was in the order placed and what was actually received
Prescribing and dispensing	Change in prescription	Perception of physician—If physicians are perceived to be professionally competent, pharmacy staff may model their behavior on physician prescribing patterns. Presence of some medical malpractice could also influence the pharmacy staff's behavior.^14^	Standardized procedure/formal communication among prescribers to adjust prescriptions during stock-outs followed Number of patients switched to another regimen due to stock-outs, then switched back to the old regimen or kept on the new regimen when the drug becomes available Changes in prescriptions recorded at the pharmacy
	Change in dispensing during stock-out	No existing indicators	Standardized procedure/formal communication among pharmacy staff regarding the amount to dispense during stock-outs followed Changes in dispensing recorded Discrepancies in what was prescribed and dispensed recorded Procedure established/followed when one of the medicines in a regimen is stocked out, and what happens to the other medicines (e.g., thrown out, given to someone else) Number of stock-outs for pediatric formulation affecting management of adult ARV stocks
	Action to ensure patient adherence	Regular pill counting^26^ Percentage of patients with full adherence to ART (i.e., no doses missed in the 3-day recall period) Average percentage of days covered by ARVs dispensed for a sample of patients for a defined period (180 days) Percentage of patients who experienced a gap in ARV availability of more than 30 days in a row during the same defined period Percentage of patients who attend on or before the day of their appointment Percentage of patients who come within 3 days of their appointment	Pill counting conducted Changes in dispensed medicines recorded	
Communication	Communication with higher-level supply chain management	No existing indicators	Perception of relationships between ARV manager/coordinator and regional offices Frequency of communication (times/month, times/year) Number of times the regional office “checks” on each pharmacy (times/month, times/year) Perception of relationships between pharmacy and central medical store Perception of support and good supervision the ARV manager thinks they receive from the regional pharmacist
	Communication with affiliated facilities	No existing indicators	Type of communications that occur between the facility and its affiliated facilities (e.g., outreach sites, baby clinics) Procedure for affiliated facilities placing orders followed Frequency that affiliated facilities place orders with the higher-level facility (times/month, times/year)	

Abbreviations: ARV, antiretroviral drug; ART, antiretroviral therapy; CMS, central medical store; RMS, regional medical store.

### Order Verification Before Submission to the Central/Regional Level (Supply Chain Area: Forecasting and Quantification)

Order verification refers to a health facility staff member rechecking the ARV requisition request (verifying calculation, order, and inventory stock) before an order is sent to the central or regional level. We noted variation across facilities within each of the 3 countries with respect to how orders are verified. In Namibia, 2 facilities have a regional pharmacist and/or senior pharmacists who verify the orders. For example, at 1 hospital, the regional pharmacist always verifies stock card records on visits, even if the senior pharmacist has verified the order and inventory check of the pharmacy assistant. In the other 2 facilities visited in Namibia, no one checks the order before it is sent. In Swaziland, 3 of the 4 hospitals have a larger pharmacy team in which a senior pharmacist supervises work. This management structure enabled verification and review of orders before submission. In Cameroon, all 4 hospitals have their orders verified by the hospital coordinator.

### Actions Taken When Stocks Are Received From CMS/RMS (Supply Chain Area: Warehousing and Inventory Management)

Actions taken when ARVs are received from the CMS/RMS refers to how and when new medications are received and added to the stock-keeping record. When stock arrives, some facilities quickly and diligently take actions, while others delay checking the physical count of medicines, opting instead to defer recording the receipt of stock and postpone storage.

In Namibia, 1 hospital prioritizes the counting, storing, and recording of new stocks upon arrival. The 3 other facilities experience delays; actions taken are less urgent or they are performed as staff becomes available. In Cameroon, 3 of 4 facilities take action when stocks are received. In 1 of these facilities, a staff member travels to the regional CAPRs to collect stocks, check physical count, and match them with the invoices. When this staff member returns to the hospital, stock cards are updated immediately with expiration dates and quantity. In 2 other facilities in Cameroon, staff members travel to the CAPRs to collect stocks and check physical counts to ensure stock cards are updated immediately upon stock arrival. In Swaziland, 2 of the 4 hospitals unpacked the new stocks when they “have time,” which can sometimes take days. The other 2 facilities unload and unpack the stocks as soon as they arrive.

### Communication With Higher-Level SCM (Supply Chain Area: Communication)

Communication by the health facilities with their regional offices refers to the dialogue and discussion around supply chain issues between individuals in the health facilities and higher-level authorities who might be MOH personnel or individuals at the CMS. In Namibia, communication with higher-level regional management in 2 facilities is reported to be quite frequent (daily communication with the regional pharmacist). The other 2 facilities in Namibia report poor communication in that the regional pharmacist “never” visits the facility.

In Cameroon, 1 facility contacts the CAPR through the hospital coordinator; in another facility, the nurse directly contacts the regional office whenever a shortage occurs. The other 2 hospitals both call their respective CAPRs on a monthly basis to check their stock levels prior to placing an order for ARVs.

Communication with higher-level SCM does not take place in Swaziland because there is no regional pharmacist; however, key informants indicated in interviews that having a regional pharmacist would be helpful. Communication with CMS is done on a frequent basis by all facilities, but the CMS is consistently slow to respond to facility requests. Therefore, health facilities at times use other sources to obtain the medicines they need, such as reaching out to donors or other health facilities to inquire about stock and delivery availability.

### Communication With Affiliated Facilities (Supply Chain Area: Communication)

Communication with the affiliated facilities refers to the dialogue and discussion of supply chain issues with other ARV clinics, other health posts, or other health personnel such as the physician who comes for outreach visits. Most facilities in Namibia reported the communication with their affiliated facilities is strong, which assists in maintaining appropriate stock levels and orders at the affiliated facilities. In Swaziland, 2 facilities reported having positive communication with their affiliated clinics, while 2 other facilities expressed concern with their clinics due to a backlog of data and nonsubmission of orders on a timely and necessary basis. No trends were reported for Cameroon because there are no affiliated facilities.

### Change in Prescription and Dispensing During Stock-Out and Action to Ensure Patient Adherence (Supply Chain Function Area: Prescribing and Dispensing)

The results highlight 3 key themes in prescribing and dispensing that are noteworthy: change in ARV prescription during stock-out, change in dispensing of ARVs during stock-out, and actions to ensure patient adherence (e.g., pill count). This section summarizes some of the trends within each country with regard to changing a patient's ARV regimen and changing dispensing practices during periods of stock-out.

In all 3 countries, each of these prescribing and dispensing practices frequently occurs in all facilities. For example, 1 facility reports changing the ARV formulation for children if there is a stock-out of a particular formulation. If the child is currently using an ARV in tablet form but the facility has a stock-out, the physician will prescribe the syrup formula instead. There are also reports of modifying the duration of the prescription. For example, if a facility is short-stocked on an ARV medication and the physician is planning to travel to reach clinics located at a further distance, the physician prescribes only 1 month, instead of the regular 2 months of ARVs. The patient must then return to the facility in 1 month, which could potentially have a negative impact on adherence. Facilities also reported changing patients to different first-line or second-line regimens that are available when a shortage occurs. However, the patient often does not resume the original regimen when the ARV is back in stock and available. In summary, patients continue with the new first- or second-line regimen unless clinical side effects are present. These regimen changes can affect consumption patterns, forecasting, and procurement and significantly contribute to consumption pattern oscillations and a lack of predictability.

### Facility-Level Practices and Behaviors With Existing and New Indicators

Existing literature and tools contain a number of indicators for forecasting and quantification and warehousing and storage.^41^ However, fewer standardized indicators to measure how facility-level behaviors and practices can affect SCM exist for prescribing and dispensing practices and communication with internal and external teams.^7^

Table 4 summarizes the mapping exercise for 7 of the 30 practices and behaviors associated with ARV SCM that were described previously, and maps each of these practices and behaviors to existing measurable indicators from the literature. For those without measurable indicators, new indicators based on country results are suggested that can potentially measure facility-level practices and behaviors and their impact on ARV SCM. Furthermore, in cases in which the qualitative data yielded important findings, new indicators are also proposed for behaviors and practices that already had some prior measures. The Supplement summarizes the mapping for all 30 practices and behaviors.

## DISCUSSION

The results of this study are derived from focus group discussions with key personnel at the hospitals and semistructured interviews with key informants consisting of higher-level health system players. The results identify 30 practices and behaviors that may affect ARV SCM, focusing on several key areas that are associated with improved SCM: order verification, actions taken when ARV stock is received, changes in prescribing and dispensing during stock-out, actions to ensure patient adherence, and communication with other affiliated facilities and higher-level SCM. We developed measurable indicators for future research, focusing on practices and behaviors related to prescribing and dispensing, communication, and human resources. With a focus on ARVs, the study results provide insight into current SCM at the facility level in Cameroon, Namibia, and Swaziland, and we make recommendations based on the findings that are considered best practices.

The results of this study identify 30 practices and behaviors that may affect ARV SCM.

It was reassuring that pharmacy staff in many of the hospitals where interviews were conducted routinely calculated min-max amounts of stock, but stock-outs of key ARV products were still an issue. Therefore, despite routinely performing these calculations, staff were not able to apply the results to prevent stock-outs from happening. Several factors might explain this discrepancy, including (1) a lack of understanding and ability to calculate min-max amounts of stock followed by application of the results, (2) lack of monitoring the calculations to ensure accuracy and to link the min-max amounts to the actual amount of stock on the shelf, (3) improperly filled orders, and (4) other miscellaneous reasons related to stock assessments. A number of other, broader contextual factors were present, including orders being placed but not filled. The current study focused on practices and behaviors at the facility level, and future research will need to incorporate additional measures and analysis of upstream contextual factors.

The findings also indicate that for some supply chain functions (forecasting and quantification and warehousing and inventory management), national guidelines and/or best practices need to be standardized and disseminated to all facilities. More specifically, information from the qualitative interviews and focus groups suggests that trainings using standardized guidelines can strengthen the following practices and behaviors: order verification, redistribution of stock, emergency orders, control and access to stock, location and condition of storage, and where ARVs are stored. Trainings should be available for all facilities and not dependent on their distance from a regional office. For example, the process of redistributing stock between facilities and requesting an emergency order was extremely varied and should be reviewed to determine best practices to be communicated to all facilities. Currently, these 2 behaviors frequently occur together and are not standardized, and redistribution often occurs between facilities using informal processes. Since emergency orders are often sent to the central level at the same time that redistribution is happening at the lower level, the central level is unaware of what has been redistributed and where, which is a broader system design and reporting issue.

Some supply chain functions need standardized national guidelines and/or best practices for dissemination to all facilities.

The impact of prescribing and dispensing practices needs to be understood clinically to determine if any of the practices and behaviors identified in this study have a negative impact on the health outcomes of the patient. There is a lack of published literature and academic understanding exactly how and to what extent these practices affect patient health. For example, changing a regimen due to a stock-out is likely to affect adherence, may cause adverse drug reactions, and could pose risks for treatment resistance. Prescribers and dispensers frequently need to manage the reality of frequent ARV stock-outs and therefore must adjust patient doses and regimens; communication between providers and facilities about these changes is minimal, and a consistent method to record this critical information is lacking. At the time of the study, Swaziland was the only country that had an electronic patient record, including prescriptions, linked with a new pharmacy electronic system. However, implementation and linking of the 2 systems had some issues, causing backlog of requests. Namibia had an electronic dispensing tool, but it was not linked with clinical records. Health facilities are often on their own in dealing with shortages and stock-outs since there are no national standard operating procedures (SOPs) or standardized prescribing guidelines specific to these emergency issues.

While the importance of communication has been identified as a key aspect of appropriate management in health care in general, little research has investigated how better communication could be linked to SCM. Five different behaviors are linked to communications, including communication within the pharmacy team; communication within the facility; communication with higher-level hospital/clinic executives; communication with higher-level supply chain (regional and central medical stores); and communication with affiliated facilities.^7^ Communication within the facility, communication with affiliated facilities, and communication with higher-level SCM offices were newly identified practices and behaviors with potential indicators to be field tested.

### Recommendations

Based on our findings, we propose recommendations to improve SCM in 4 main facility-level areas.

- **Understanding and monitoring of routine min-max calculations.** Pharmacy staff should be monitored to perform the min-max calculations. This monitoring can happen within the facility by another colleague, such as a hospital coordinator, or by a higher-level regional pharmacist. Many hospitals relied on their regional pharmacist for periodic visits to check on specific practices and behaviors, reporting that the monitoring improved their SCM. Facilities in regions that did not have a regional pharmacist reported that having one would be helpful in such monitoring activities.
- **Development of national guidelines and norms.** Best practices and guidelines should be developed and monitored at the facility level for order verification, redistribution of stock, emergency orders, control and access to stock, location and condition of storage, and where ARVs are stored.
- **Understanding the impact of prescribing and dispensing practices on health outcomes.** Prescribers and dispensing staff would certainly benefit from guidelines, SOPs, and trainings on ARV regimen switches and managing shortages. Additional research is needed to understand the following: how prescribing and dispensing practices change during shortages and stock-outs; the frequency of regimen changes practiced in the country; and the impact of regimen changes on health outcomes including adherence and resistance.
- **The importance of communication.** Communication has been more frequently measured and studied in hospital management, business, and organizational fields; however, how different types of communication can affect SCM has not been thoroughly examined or measured with standardized indicators. This gap holds true at the facility, regional, and central levels.^7^

Our recommendations are most likely applicable to other countries around the world that are experiencing similar difficulties with their own ARV supply chains. Further research, particularly field-based, pilot testing of the new, potential indicators for communication, human resources, and prescribing and dispensing identified in this study, is needed to understand their impact on SCM. Once these potential indicators are piloted for validity and reliability, causal study designs can be developed to test which practices and behaviors have a direct, measurable impact on facility-level SCM.

### Limitations

Our study yielded some interesting findings, but it had some limitations. First, it was limited to selecting PEPFAR-supported countries based on availability and preparedness of the country teams. Secondly, the facility selection indicators varied across all 3 countries using information from country experts and available in-country data. Due to this wide variation in selection criteria for facilities across the countries, cross-country or intracountry comparisons or causal conclusions could not be made. Consequently, for each generalized result given above, a country-specific example is provided. While this is a major limitation, which is exacerbated by the sample size, it provides a baseline and unique opportunity to investigate these associations in more detail within each country. Furthermore, our study did not consider how gender, ethnicity, socioeconomic status, or other power dynamics may influence the lens through which the key informants viewed the issues. Finally, the relationship between facility- and central-level indicators (i.e., CMSs) is not explored in this article; however, improvements in facility-level practices and behaviors should logically lead to better SCM at the central level.

Due to these limitations, no cross-country or intra-country comparisons or conclusions could be made about potential linkages between the facility-level practices and behaviors identified and SCM outcomes.

## CONCLUSIONS

This study identifies gaps in the measurement of facility-level practices and behaviors in the ARV supply chain. The results identify 30 facility-level practices and behaviors associated with SCM; 15 of the 30 do not have any existing indicators for measurement from literature and research. Potential new indicators are proposed based upon the analysis of the in-country qualitative results.

Improved practices and behaviors are recommended for implementation, such as offering trainings, developing SOPs and national guidelines related to communication with internal and external staff, developing new guidelines on how to adjust prescribing and dispensing patterns during shortages or stock-outs, and limiting access to ARVs in storage to selected staff at the facility. The study results provide insight into the way that ARV supply chains are currently managed at the facility level in 3 countries of sub-Saharan Africa. The results may serve as the basis for additional research, both within and across countries, to examine how improving specific behavior and practices affects SCM.

## Notes

**Peer Reviewed**

**Funding:** This article was made possible by the generous support of the American people through USAID, under the terms of cooperative agreement number AID-OAA-A-11-00021. The authors led the study design, data collection, analysis, and interpretation of the data as well as the writing of the manuscript. The funding entities, USAID and MSH, provided input into the study design and overall research concept.
